# Assessing the differential impact of chronic CMV and treated HIV infection on CD8+ T-cell differentiation in a matched cohort study: is CMV the key?

**DOI:** 10.1186/s12981-021-00361-z

**Published:** 2021-06-30

**Authors:** Matthias C. Mueller, Winfried V. Kern, Susanne Usadel, Marie-Christin Pauly, Toni Cathomen, Ulrich Salzer

**Affiliations:** 1 Division of Infectious Diseases, Department of Medicine II, Medical Center–University of Freiburg, Faculty of Medicine, University of Freiburg, Hugstetter Straße 55, 79106 Freiburg, Germany; 2 Department of Infection Medicine, Medical Service Centre Clotten, Freiburg, Germany; 3 Institute for Transfusion Medicine and Gene Therapy, Medical Center–University of Freiburg, Faculty of Medicine, University of Freiburg, Freiburg, Germany; 4 Department of Rheumatology and Clinical Immunology, Medical Center–University of Freiburg, Faculty of Medicine, University of Freiburg, Freiburg, Germany

**Keywords:** HIV, CMV, T-cell senescence, CD163, CRP, Inflammation

## Abstract

**Background:**

Cytomegalovirus (CMV) infection is one of the main driving forces of T-cell senescence in the general population, whereas its differential impact in people living with HIV (PLWH) is less well characterized. The study explores the effect of latent CMV infection on T-cell subsets, monocyte/macrophages activation markers, and CRP in PLWH on long-term ART.

**Methods:**

Cross-sectional cohort study including PLWH on long-term suppressive ART. Individuals of 4 groups (HIV+CMV−, HIV+CMV+, HIV−CMV+, and HIV−CMV−) were matched 1:1:1:1 for age and sex. Immunophenotyping of lymphocyte and T-cell subsets by multicolor flow cytometry was performed in fresh blood samples collected from patients and healthy donors.

**Results:**

Both, latent CMV and treated HIV infection were associated with an expansion of CD8 T cells, a reduced CD4/CD8 ratio, and with CD8 T-cell activation with a cumulative effect in CMV/HIV-coinfected individuals. CMV was associated with elevated numbers of late effector and terminally differentiated CD8 T-cells. Compared to CMV monoinfection, CMV/HIV coinfection showed to be associated with lower proportion of CD28−CD8+ T cells expressing CD57 suggesting that HIV preferentially expands CD28−CD57−CD8+ T cells and impedes terminal differentiation of CD28−CD8+ T cells. We could not show any association between HIV or CMV infection status and concentration of CRP and CD163.

**Conclusions:**

CMV infection is associated with phenotypic signs of T-cell senescence, promoting exacerbation and persistence of alterations of the T-cell compartment in PLWH on effective ART, which are associated with adverse clinical outcomes and may be an attractive target for therapeutic interventions.

**Supplementary Information:**

The online version contains supplementary material available at 10.1186/s12981-021-00361-z.

## Background

Antiretroviral therapy (ART) led to a remarkable improvement in long-term life-expectancy of people living with HIV (PLWH) [1]. Despite treatment-mediated continuous suppression of HIV replication and consecutive restoration of CD4 cell count, PLWH retain a higher risk for death and aging-related disorders including cardiovascular, renal, liver, neurologic, and bone disease [2]. Because HIV induces immunologic dysfunctions showing similarities to those observed in elderly populations, it has been hypothesized that HIV-induced accelerated aging of the immune system (immune senescence) could mediate these risks.

T-cell senescence, whether driven by aging and/or by chronic antigenic stimulation from pathogens such as HIV or cytomegalovirus (CMV), is typically characterized by T-cell activation, inversion of the CD4/CD8 ratio due to low CD4 T-cell counts and accumulation of terminally differentiated CD8 T cells with shortened telomeres, a decrease in the costimulatory molecule CD28, and increased expression of CD57, a marker of proliferative history and poor proliferative capacity [3, 4].

Markers of inflammation like c-reactive protein (CRP) and of monocyte/macrophage activation, like soluble CD14 (sCD14) and soluble CD163 (sCD163), have been shown to be elevated in HIV infection and to be associated with the risk for non-AIDS-defining morbidity, especially with cardiovascular disease [5–11].

In PLWH, ART improves many of these HIV-induced immunologic alterations, but the effect is incomplete, and markers of immune activation, inflammation and T-cell differentiation remain altered. The mechanism inducing residual immune dysfunction is complex and multifactorial in origin. Chronic coinfection with CMV has been identified to strongly contribute to persistent immune activation, to be associated with lower CD4/CD8 T-cell ratios, with reduced expression of CD28 and increased expression of CD57 in PLWH people with HIV on long term ART [3, 12–14].

Differences in alterations of T-cell compartments between HIV infection on one side and CMV monoinfection and aging on the other side may exist. In a study of Lee and colleagues, CMV infection and aging were associated with high proportions CD28−CD8+ T-cells expressing CD57, whereas PLWH showed an enrichment of less well-differentiated transitional CD8+ T cells and abnormally low proportions of CD28−CD8+ T cells expressing CD57. In a consecutive study, the authors could show that low proportions of CD28−CD8+ T cells expressing CD57 strongly predicted increased all-cause mortality in PLWH under ART [15, 16]. These studies however did not assess the effect of CMV coinfection on the magnitude of CD57 expression of CD28−CD8+ T cells and on clinical outcome.

Equally, many other studies assessing the HIV-induced effects on immune senescence did not control for CMV coinfection and/or did not employ HIV-negative individuals, making it impossible to discriminate the effects of CMV and HIV on the immune dysfunction perceived in PLWH on long term ART [3, 12–14, 17–19]. CMV prevalence in the general population increases with age and is 55% among blood donors in Germany, whereas the CMV prevalence in HIV cohorts generally is about 90% independent of age [20, 21].

To evaluate the differential effect of CMV and HIV infection on markers of immune dysfunction, we performed a cohort study matching for sex and age CMV-negative and -positive individuals with and without HIV infection.

## Methods

### Ethics consideration

The study was approved by the institutional ethics review board of the medical center of the University of Freiburg (No.: 317/15, Date: 31 July 2015). All PLWH were recruited at the HIV Centre Freiburg and provided written informed consent. Blood donors provided informed consent for the scientific use of surplus portions of their blood donation.

### Study design/setting

This cross-sectional cohort study was carried out at the HIV Centre Freiburg, Germany, caring for about 800 PLWH. Patients were enrolled between November 26, 2015 and November 23, 2016. Because CMV seronegative PLWH are rare, patient records of PLWH attending the HIV Centre Freiburg were screened for CMV seronegative patients. Blood samples were taken at enrollment of patients and CMV serostatus was reassessed using a CMV immunoglobulin G (IgG) assay (Abbott Architect CMV IgG CLIA, Abbott Diagnostics, Wiesbaden, Germany). HIV-infected CMV-seronegative patients were then matched 1:1:1:1 for sex and age with HIV-infected, CMV-seropositive patients and with HIV-uninfected, CMV-seropositive and CMV-seronegative blood donors from the Blood Donation Center Freiburg.

HIV-infected patients were eligible when they were on ART with ≥ 12 months of HIV RNA < 20 cp/ml. Exclusion criteria for HIV-infected patients were active Hepatitis B or Hepatitis C, untreated sexual transmitted disease (syphilis, gonorrhea, and *Chlamydia trachomatis* infection), previous or current CMV-related organ diseases, organ transplantation, as well as cancer or treatment of cancer, autoimmune disease with or without use of immunosuppressive or immunomodulant drugs in the previous 5 years.

### Study diagnostic procedures

#### Lymphocyte and T-cell subpopulations phenotyping

Phenotyping of T-, B- and NK cells within the lymphocyte population as well as T-cell subpopulations was performed by a whole blood staining lyse-no wash protocol (Optilyse B, Beckman-Coulter) using six colour flow cytometry with fluorochrome-conjugated antibodies as listed in Additional file 1: Table S1.

Antibody labelled cells were analyzed by flow cytometry (Navios; Beckman Coulter). Absolute cell counts were calculated using a two platform method with leucocyte and lymphocyte counts determined by a hemocytometer. Flow cytometric data analysis was performed with the help of Kaluza Software 1.5a (Beckman Coulter). Representative gating strategies for analysed populations are shown in Additional file 2: Figure S1 and Additional file 3: Figure S2.

#### sCD163 and CRP analysis

sCD163 concentrations in cryopreserved plasma samples were quantified by using a commercially available ELISA kit (Quantikine, R&D Systems, Minneapolis, MN, United States of America). All samples were tested in duplicate. hsCRP concentrations in cryopreserved plasma samples were quantified by using the standard immunoturbidometric assay on the COBAS® INTEGRA system (Roche COBAS INTEGRA, Roche Diagnostics, Basel Switzerland).

### Statistical analysis

A Kruskal–Wallis test was conducted to determine if levels of CRP and sCD163 were statistically different between the four cohorts. Spearman’s rank correlation coefficient was calculated to determine associations between variables. For pairwise statistical analysis of flow cytometric two sided Wilcoxon matched-pairs signed rank test (analyzed data showed a non-Gaussian distribution) was performed for cell counts of individual cell populations. Normality testing was done using the D'Agostino & Pearson normality test. Statistical results were calculated with the of Graphpad Prism Software version 8 (Graphpad Software, San Diego, USA).

## Results

A total of 92 patients were included in the study, forming 23 sets with each 4 matched individuals. Median age was 49.4 years [interquartile range (IQR), 44.3–54.3] and 30.4% of participants were women without differences between the 4 groups (Table 1). Median time of HIV RNA below limit of detection was 64 months (IQR, 20–96) in HIV+CMV+ and 62 months (IQR, 15–108) in HIV+CMV− patients.

**Table 1:** Baseline characteristics of participating patients matched for sex and age recruited from a HIV cohort between November 2015 and November 2016

	Total	HIV+		HIV−	
		CMV+	CMV−	CMV+	CMV−
n	92	23	23	23	23
Female, n (%)	28 (30.4)	7 (30.4)	7 (30.4)	7 (30.4)	7 (30.4)
Age (y), median (IQR)	49.4 (44.3–54.3)	50.1 (44.3–54.3)	48.6 (44.3–54.0)	49.4 (44.1–55.4)	49.5 (45.2–55.0)
Time since HIV infection (y), median (IQR)	14.5 (6.6–21.0)	12.5 (6.6–19.7)	17.1 (6.5–23.1)	NA	NA
CD4 nadir (cells/µl), median (IQR)	190.5 (95.0–255.0)	197.0 (95.0–257.0)	173.5 (95.0–223.0)	NA	NA
VL < LoD (months), median (IQR)	63.0 (20.0–99.0)	64.0 (20.0–96.0)	62.0 (15.0–108.0)	NA	NA
CD4, (cells/µl), median (IQR)	NA	469.5 (364.5–635.3)	564.0 (380.8–727.3)	825.0 (584.8–1054.0)	758.0 (593.0–1045.0)

*VL* viral load, *LoD* limit of detection, *y* year, *ART* antiretroviral therapy, *CMV* cytomegalovirus, *NA* not applicable, *IQR* interquartile range

### Lymphocyte and T-cell subpopulations phenotyping

**Fig. 1:**
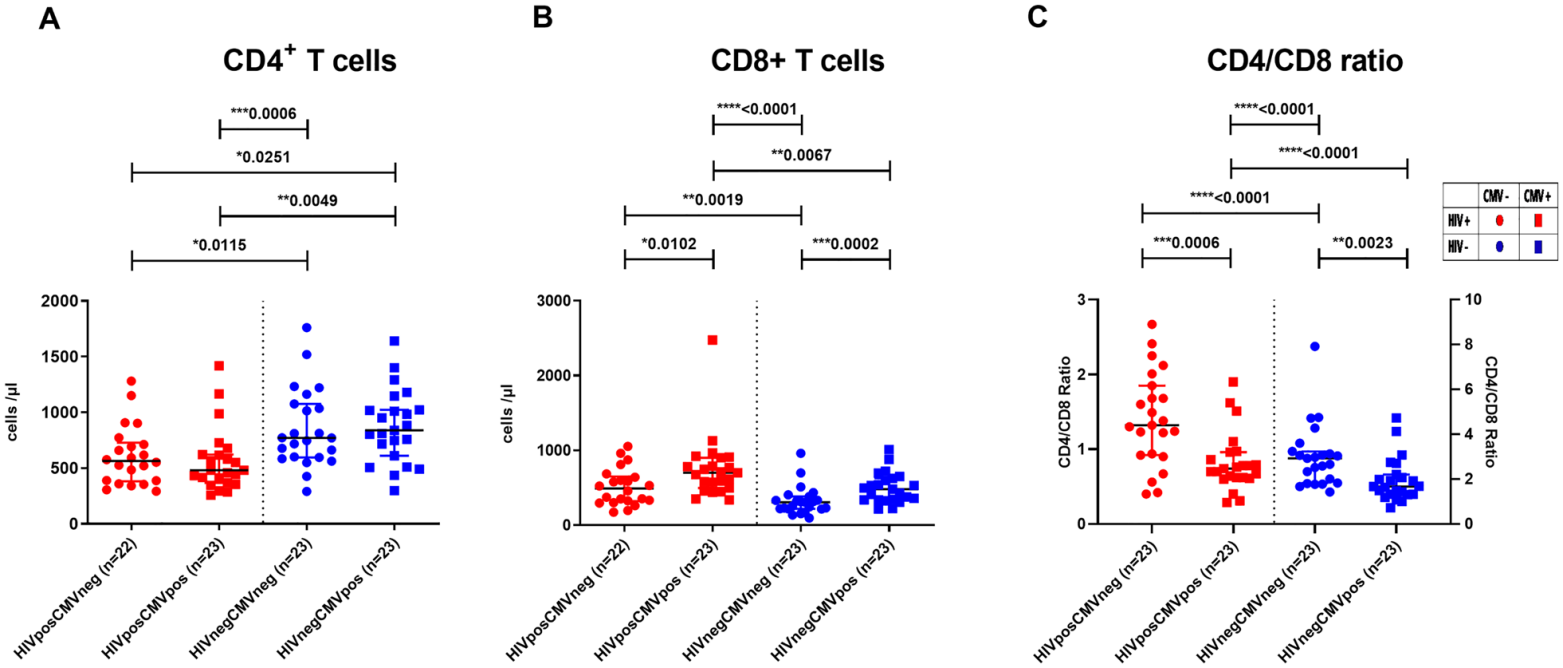
Lymphocyte subsets and activated T cells. **A** Absolute counts of CD4+ T cells/µl. **B** absolute counts of CD8+ T cells/µl. **C** CD4/CD8 ratio. Black horizontal lines correspond to the median and errors bars show the interquartile ranges. Red circles: HIV positive and CMV negative individual; red squares: HIV positive and CMV positive individual; blue circles: HIV negative and CMV negative individual; blue squares: HIV negative and CMV positive individual

As expected, lymphocyte subpopulation analysis revealed lower amounts of CD4+ T cells in HIV infected individuals (median^HIV+CMV+^ = 469.5/µl versus median^HIV−CMV+^ = 825.0/µl; p = 0.0049 and median^HIV+CMV−^ = 564/µl versus median^HIV−CMV−^ = 758.0/µl; p = 0.0115) but there was no significant difference between the CMV+ and CMV− groups both in PLWH and healthy controls (Fig. 1A). The further analysis revealed higher absolute numbers of CD8+ T cells in CMV+ patients both in the HIV− and HIV+ groups (median^HIV+CMV+^ = 700/µl versus median^HIV+CMV−^ = 491.5/µl, p = 0.0102 and median^HIV−CMV+^ = 482.0/µl versus median^HIV−CMV−^ = 306/µl, p = 0.0002; Fig. 1B) and a significantly lower CD4/CD8 ratio in CMV+ subjects both in the HIV− and HIV+ groups (median^HIV+CMV+^  = 0.74 versus median^HIV+CMV−^ = 1.32, p = 0.0006 and median^HIV−CMV+^ = 1.67 versus median^HIV−CMV−^ = 2.93; p = 0.0023, Fig. 1C).

### T-cell subpopulations phenotyping

**Fig. 2:**
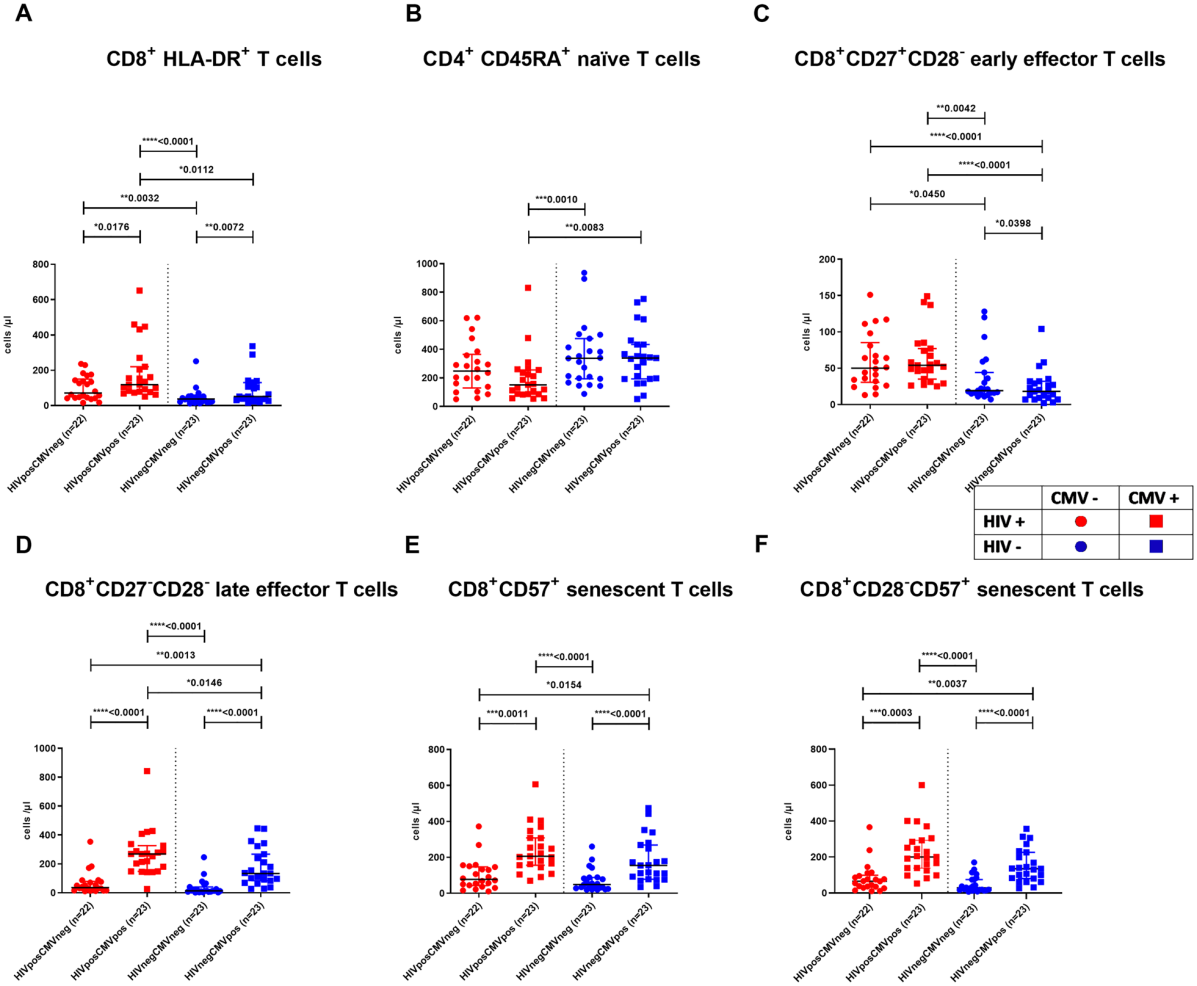
CD4+ and CD8+ T-cell subpopulations. **A** Absolute counts of CD8+ HLA-DR+ T cells/µl. **B** Absolute counts of CD4+CD45RA+ naïve T cells/µl. **C** absolute counts of CD8+CD28+ CD27− early effector T cells/µl. **D** Absolute counts CD8+CD28−CD27− late effector T cells/µl. **E** Absolute counts CD8+CD57+ terminally differentiated T cells/µl. **F** Absolute counts CD8+CD28−CD57+ terminally differentiated T cells/µl. Black horizontal lines correspond to the median and errors bars show the interquartile ranges. Red circles: HIV positive and CMV negative individual; Red squares: HIV positive and CMV positive individual; blue circles: HIV negative and CMV negative individual; blue squares: HIV negative and CMV positive individual

For analysis of T-cell activation markers, HLA-DR was counterstained and analyzed on CD3+, CD4+ and CD8+ T cells. This showed higher absolute numbers of CD8+HLA−DR+ T cells in CMV+ patients both in the HIV− and HIV+ groups (median^HIV+CMV+^ = 118/µl versus median^HIV+CMV−^ = 71/µl, p = 0.0176 and median^HIV−CMV+^ = 51/µl versus median^HIV−CMV−^ = 37/µl, p = 0.0072; Fig. 2A).

Naïve CD4+CD45RA+ T cells were lower in absolute numbers in HIV+CMV+ patients when compared to CMV+ healthy controls (median^HIV+CMV+^ = 151/µl versus median^HIV−CMV+^ = 339/µl, p = 0.0083) (Fig. 2B). The expanded CD8+ T-cell subset of CMV+ subjects was enriched for CD8+CD27−CD28− early and late effector cells in HIV+ and HIV− groups (median^HIV+CMV+^ = 269/µl versus median^HIV+CMV−^ = 36.5/µl, p < 0.0001 and median^HIV−CMV+^ = 134/µl versus median^HIV−CMV−^ = 14/µl; p < 0.0001) (Fig. 2C, D) and CD8+CD57+ terminally differentiated CD8 T cells (median^HIV+CMV+^ = 206/µl versus median^HIV+CMV−^ = 77.5/µl; p = 0.0011 and median^HIV−CMV+^ = 155/µl versus median^HIV−CMV−^ = 49/µl, p < 0.0001) (Fig. 2E). Within the CD8+CD28− T-cell subset the increase of CD57+ cells was even more significant (median^HIV+CMV+^ = 200/µl versus median^HIV+CMV−^ = 65.5/µl; p = 0.0003 and median^HIV−CMV+^ = 135/µl versus median^HIV−CMV−^ = 29/µl, p < 0.0001) (Fig. 2F).

### Markers of inflammation and monocyte activation

**Fig. 3:**
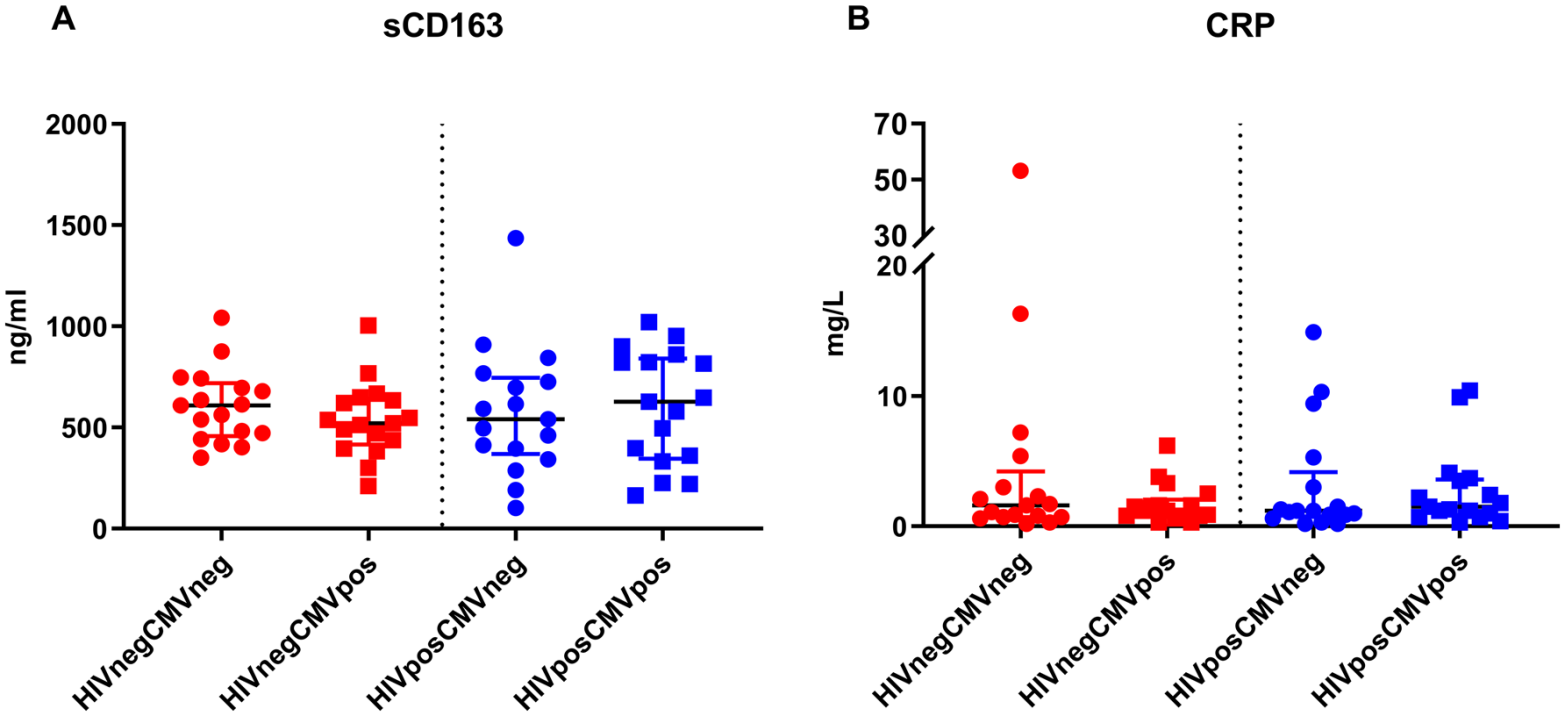
Serum concentrations of inflammation markers. **A** Serum levels of soluble CD 163 (sCD163) and **B** C-reactive protein (CRP) in CMV seropositive and seronegative HIV-infected patients on long term ART and HIV-naïve control group. Black horizontal lines correspond to the median and errors bars show the interquartile ranges. Red cricles: HIV positive and CMV negative individual; red squares: HIV positive and CMV positive individual; blue circles: HIV negative and CMV negative individual; blue squares: HIV negative and CMV positive individual

In the 17 quadruples with available sCD163 concentration measurement of all four patients the median sCD163 concentration was 555 ng/ml (IQR, 408.0–733.5 ng/ml), without differences between the four subgroups [HIV+CMV+: 628 ng/ml (IQR, 360.0–822.0 ng/ml), HIV+CMV−: 540 ng/ml (IQR, 396.0–725.0 ng/ml), HIV−CMV+: 520 ng/ml (IQR, 437.0–634.0 ng/ml) and HIV−CMV−: 609 ng/ml (IQR, 472.0–695.0 ng/ml), p = 0.9208] (Fig. 3). Correlation analysis showed that sCD163 levels were positively correlated with age in the group of CMV+HIV+ (r = 0.49, p = 0.020) and in CMV+HIV− with borderline statistical significance (r = 0.63, p = 0.062) but not in CMV− subgroups (CMV−HIV−: r = − 0.146, p = 0.529; CMV−HIV+: r = 0.257, p = 0.274). We could neither detect an association between sCD163 levels and duration of HIV infection (CMV+HIV+: r = 0.311, p = 0.159; CMV−HIV+: r = 0.298, p = 0.202) nor with CD4 Nadir (CMV+HIV+: r = − 0.181, p = 0.593; CMV−HIV+: r = 0.128, p = 0.709) or current CD4 cell count (CMV+HIV+: r = − 0.018, p = 0.9377; CMV−HIV+: r = 0.104, p = 0.6633).

In the 17 quadruples with available CRP concentration measurement of all four individuals the median CRP concentration was 1.2 mg/l (IQR, 0.7–3.0 ng/ml), without differences between the four subgroups [HIV+CMV+: 1.6 mg/l (IQR, 1.0–3.5 mg/l), HIV+CMV−: 1.2 mg/l (IQR, 0.7–2.25 mg/l), HIV−CMV+: 1.2 mg/l (IQR, 0.8–2.5 mg/l) and HIV−CMV−: 1.1 mg/l (IQR, 0.7–3.0 mg/l), p = 0.684] (Fig. 3).

## Discussion

To our knowledge, this is the first study showing the differential impact of CMV infection and treated HIV infection on T-cell subpopulations.

### Effect of infection with HIV and/or CMV on lymphocyte subsets, T-cell activation and T-cell subpopulations

In our four cohorts matched for sex and age, we found that treated HIV monoinfection and isolated CMV infection are associated with similar levels of activation of CD8 T cells with a cumulative effect in PLWH with long term ART and CMV coinfection resulting in high numbers of activated CD8 T cells. Wittkop and colleagues similarly demonstrated that CMV infection is associated with activation of CD8 T cells in treated HIV-infected individuals but they were not able to show the differential effect of HIV and CMV infection due to the absence of a HIV-naïve control group [3]. Here we demonstrate that CMV coinfection may be a major but not exclusive cause of residual T-cell activation in treated HIV-infected individuals. Other factors that are proposed to contribute to T-cell activation in this setting are age, microbial translocation, and residual replication of HIV in latently infected cells [22].

Both monoinfection with CMV and effectively treated HIV-monoinfection were associated with expansion of CD8 T cells to comparable levels, resulting in a cumulative effect in people coinfected with HIV/CMV. Greater CD8 T-cell inflation in combination with HIV-induced reduction of CD4 T-cell counts led to a CD4/CD8 ratio below 1 in CMV coinfected patients. Patients with treated HIV-monoinfection or isolated CMV-infection displayed similar reduction of CD4/CD8 ratios compared to CMV−/HIV-naïve individuals. Freeman and colleagues showed similar results concerning the effect of CMV coinfection on CD8 expansion and CD4/CD8 ratio in treated HIV-positive individuals [14]. Their incapability to attribute CD8 T-cell expansion to treated HIV-monoinfection and to demonstrate an effect of CMV infection on CD4/CD8 ratio in HIV-naïve patients may be probably due to missed matching for CMV status, sex and age of HIV-negative controls [14, 23]. Indeed, previous studies demonstrated a clear effect of CMV infection on the frequency of CD8 T cells and on the CD4/CD8 ratio in HIV-negative, especially elderly individuals [23, 24].

The effect of infection with HIV and/or CMV on late effector CD8 T cells and terminally differentiated CD8 T cells showed similar patterns. CMV infection was associated with high numbers of late effector and terminally differentiated CD8 T cells in HIV-naïve and PLWH on ART, showing a stronger effect of CMV infection on the levels of late effector T cells in treated HIV-positive than in HIV-naïve individuals. Whereas the absolute numbers of late effector and terminally differentiated T cells in CMV-monoinfected patients were clearly raised, the effect of treated HIV-monoinfection compared to CMV/HIV naïve study participants showed no statistically significant effect. Previous studies showed that proportions of late effector and terminally differentiated CD8 T cells strongly decreased after initiation of ART but remained significantly higher compared to HIV-negative individuals after up to 144 weeks of treatment [17, 25]. Because the studies were controlled for age and sex, but not for CMV serostatus, it may be assumed that the persistent alterations of the distribution of late effector and terminally differentiated CD8 T cells is probably due to the more pronounced effect of CMV infection on late effector CD8 T cells in individuals with HIV and the higher prevalence of CMV infection in HIV-cohorts compared to the general population. Thus, the persistence of elevated levels of late effector and terminally differentiated T cells in PLWH under effective ART is probably not a residual effect of HIV-infection but mainly attributable to CMV coinfection.

Our results are in line with previous data showing an association between CMV infection and high proportion of CD28−CD8+ T cells expressing CD57 in HIV-naïve individuals [23]. In concordance to the results of the study of Lee and colleagues restricted to patients with confirmed latent CMV infection, we show that treated HIV infection compared to CMV-monoinfection is associated with lower proportion of CD28−CD8+ T cells expressing CD57 [15]. Higher absolute numbers of CD28−CD57+CD8+ T cells were due to an expansion of CD28−CD8+ populations in HIV−/CMV-coinfected individuals, suggesting that HIV preferentially expands CD28−CD57−CD8+ T cells and impedes the terminal differentiation of CD28−CD8+ T cells. This effect of HIV on the differentiation process of CD8 T cells may explain our findings that frequencies of CD28−CD8+ T cells expressing CD57 were comparable in both cohorts of HIV-infected individuals regardless of CMV serostatus, suggesting that HIV infection may have a modulatory effect on the T-cell response to CMV infection.

These alterations of the T-cell compartment may have consequences in the clinical outcome. In the HIV‐negative population, a low CD4/CD8 ratio has been associated with near‐term mortality in the elderly [26]. In ART-treated HIV-infected individuals, expansion of CD8 T cells and inversion of CD4/CD8 ratio has been linked to an increased morbidity and mortality, even in the setting of CD4 T-cell counts within the reference range [27, 28]. Associations of higher frequencies of terminally differentiated (CD28−CD57+) and activated (CD38+HLA−DR+) CD8+ T cells with subclinical carotid artery disease have been described in PLWH [29]. These markers were also used to generate a score of immune activation and senescence which showed a statistically significant association with the development of non-AIDS related morbidities in treated patients younger than 60 years [30].

It has been proposed that the HIV-induced impediment of terminal differentiation of CD28−CD8+ T cells may lead to a functional immune defect as CD57+CD8+ T cells are believed to be highly effective at killing infected cells and a high percentage of CD57+CD8+ T cells has been shown to be associated with lower HIV RNA level set-points [15, 31, 32]. Furthermore, Lee and colleagues could show that low proportions of CD28−CD8+ T cells expressing CD57+ predicts increased all-cause mortality in PLWH under ART [16].

### Effect of infection with HIV and/or CMV on markers of inflammation and monocyte/macrophages activation

In the current study, concentrations of CRP and sCD163 in PLWH were in the magnitude of those of HIV-negative individuals without effect of CMV. Previous studies agree that CRP is elevated and persists in chronically HIV-infected patients without effect of ART even after several years of treatment [8, 33, 34]. Valid data on the effect of CMV infection on CRP levels are scarce. The available evidence shows an effect of CMV infection on CRP levels in HIV-negative patients and HIV-positive individuals with and without ART [35, 36]. Concerning sCD163, previously published results of comparable cohorts showed higher concentrations of sCD163 in PLWH under long term ART and undetectable HIV RNA levels compared to age matched HIV-seronegative controls, but none of them was controlled for CMV serostatus [5, 6, 11]. Vita and colleagues found significantly higher sCD163 plasma levels in HIV/CMV coinfected compared to HIV monoinfected subjects matched for age, CD4 nadir, HIV infection duration, and viral hepatitis status [37]. Interestingly, sCD163 concentrations of patients with HIV monoinfection were comparable with those who were not infected with HIV, suggesting a driving role of chronic CMV infection in monocyte/macrophage activation in treated individuals with HIV. Levels of sCD163 were positively correlated with duration of HIV infection which could explain the absence of an effect of CMV on sCD163 levels in our study as we did not match for this variable. But in our study, we could not confirm the association of duration of HIV infection and sCD163.

A limitation of our study is the relatively small sample size. Differences which did not reach statistical significance in our study may become statistically significant in a larger sample set. Although we employed a prospective study design, some data had to be extracted from patients’ records leading to an incomplete data set in some of the items.

## Conclusions

We could show that latent CMV infection is probably the major contributor to persistent alterations in the T-cell compartment linked to adverse clinical outcomes in treated HIV infection and that HIV infection may modulate T-cell response against CMV infection. Therefore, future studies investigating immune senescence should always control for CMV serostatus.

In both HIV and CMV infection perpetual antigen presentation is the driving force of these changes in the T-cell compartment. In the setting of HIV infection it is now widely accepted that suppressive therapy of HIV replication improves parameters of immune activation, immune senescence and inflammation and reduces the incidence of non-AIDS-associated morbidities [38, 39].

In the setting of CMV infection, it has been shown that valganciclovir reduces CMV replication and CD8 T-cell activation in treated HIV-infected individuals but trials evaluating the effect of anti-CMV therapy on markers of immune senescence and the incidence on age-related morbidity are lacking [12]. Because novel, well tolerated anti-CMV therapies are now available, CMV infection may be an attractive target for therapeutic interventions in individuals with HIV on effective ART [40].

## Supplementary Information

**Additional file 1: Table S1.** Table with antibodies used for Lymphocyte and T-cell subpopulations phenotyping by flow cytometry.

**Additional file 2: Figure S1.** Gating strategy for T-cell subsets.

**Additional file 3: Figure S2.** Gating strategy for lymphocyte subsets.

## Data Availability

All data generated or analyzed during this study are included in this published article and its Additional files.

## Abbreviations

ART: Antiretroviral therapy
CMV: Cytomegalovirus
CRP: C-reactive protein
IQR: Interquartile range
PLWH: People living with HIV
sCD14: Soluble CD14
sCD163: Soluble CD163
