# Supplementary material for: Assessing the differential impact of chronic CMV and treated HIV infection on CD8+ T-cell differentiation in a matched cohort study: is CMV the key?
Source: AIDS Res Ther. 2021 Jun 30;18:37. doi: 10.1186/s12981-021-00361-z (PMC8247205; doi:10.1186/s12981-021-00361-z)
Supplement: Supplementary file 1 — Additional file 1: Table S1. Table with antibodies used for Lymphocyte and T-cell subpopulations phenotyping by flow cytometry. [file 12981_2021_361_MOESM1_ESM.pdf]

antigen	*fluorochrome*	*antibody clone*	*manufacturer*	*Population(s)*
**CD3**	BV 421	UCHT1	Biolegend	T-cells
**CD3**	Pacific Blue	UCHT1	Biolegend	T-cells
**CD4**	APC	SK3	Becton Dickinson	T-Helper cells
**CD4**	PE-Cy7	SK3	Biolegend	T-Helper cells
**CD8**	FITC	B9.11	Beckman Coulter Immunotech	cytotoxic T-cells
**CD8**	PerCP	SK1	Biolegend	cytotoxic T-cells
**CD16**	PE	3G8	Beckman Coulter Immunotech	NK cells
**CD19**	PE-Cy7	J3-119	Beckman Coulter Immunotech	B-cells
**CD27**	PE-Cy7	M-T271	Becton Dickinson	early & late CD8+ effector cells
**CD28**	PE	L293	Becton Dickinson	early & late CD8+ effector cells
**CD45**	PerCP	HI30	Biolegend	leucocytes
**CD45RA**	APC	MEM-56	Exbio	naive T-cells
**CD45RO**	PE	UCHL1	Beckman Coulter Immunotech	memory T-cells
**CD56**	PE	N901	Beckman Coulter Immunotech	NK cells
**CD57**	FITC	NC1	Beckman Coulter Immunotech	senescent CD8+ T-cells
**HLA-DR**	FITC	L243	Becton Dickinson	activated T-cells

Additional file 1: Table S1. antibodies used for Lymphocyte and T-cell subpopulations phenotyping by flow cytometry
