# Supplementary figures and images for: Assessing the differential impact of chronic CMV and treated HIV infection on CD8+ T-cell differentiation in a matched cohort study: is CMV the key?
Source: AIDS Res Ther. 2021 Jun 30;18:37. doi: 10.1186/s12981-021-00361-z (PMC8247205; doi:10.1186/s12981-021-00361-z)

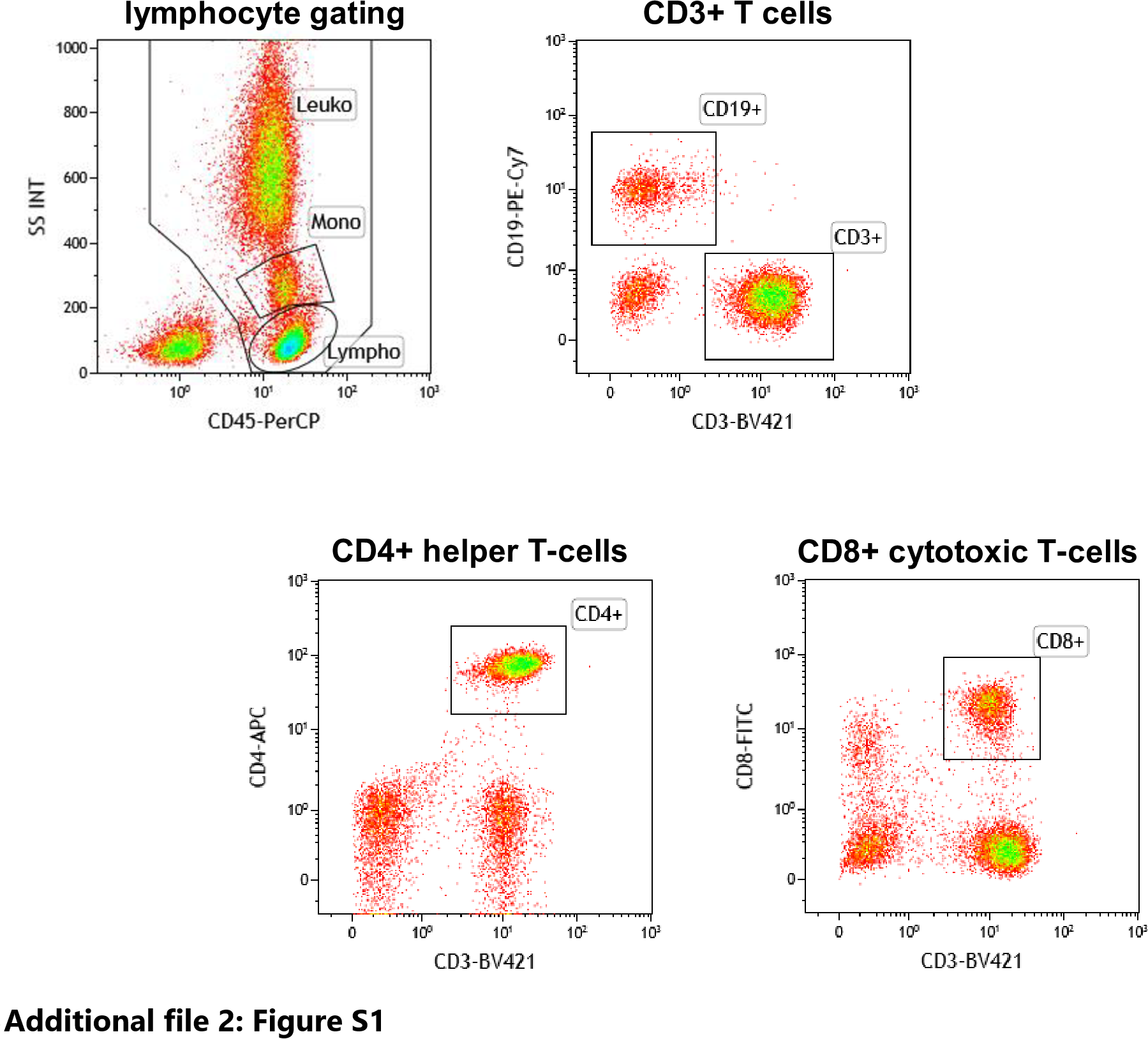

Supplement: Supplementary file 2 — Additional file 2: Figure S1. Gating strategy for T-cell subsets. [file 12981_2021_361_MOESM2_ESM.tiff]

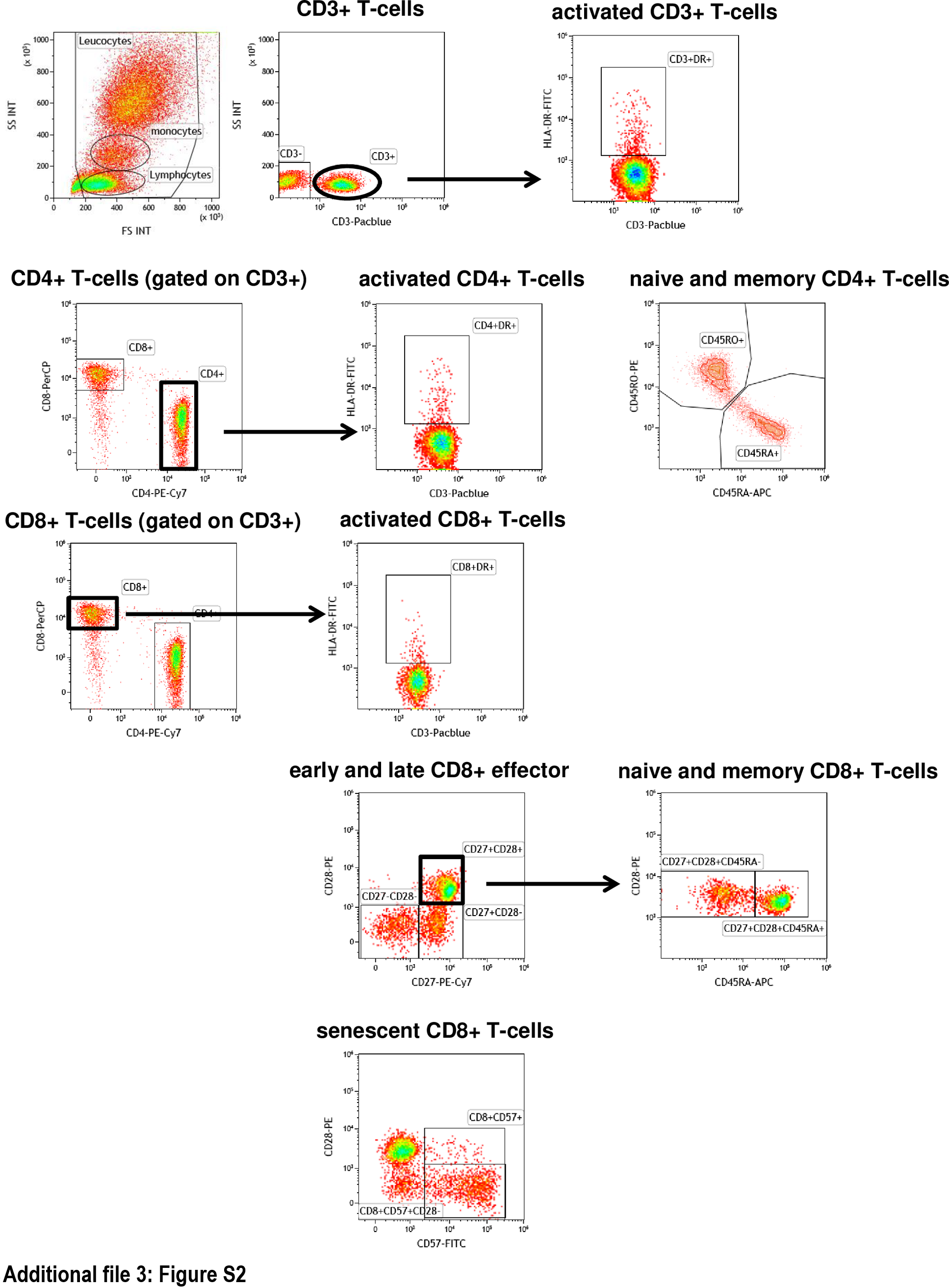

Supplement: Supplementary file 3 — Additional file 3: Figure S2. Gating strategy for lymphocyte subsets. [file 12981_2021_361_MOESM3_ESM.tiff]
